# Efficacy, safety, and tolerability of imepitoin in dogs with newly diagnosed epilepsy in a randomized controlled clinical study with long-term follow up

**DOI:** 10.1186/s12917-015-0548-9

**Published:** 2015-09-02

**Authors:** Chris Rundfeldt, Andrea Tipold, Wolfgang Löscher

**Affiliations:** Drug-Consulting Network, 01445 Coswig, Germany; Department of Small Animal Medicine and Surgery, University of Veterinary Medicine, Hannover, Germany; Center for Systems Neuroscience, 30559 Hannover, Germany; Department of Pharmacology, Toxicology and Pharmacy, University of Veterinary Medicine Hannover, 30559 Hannover, Germany

**Keywords:** Idiopathic epilepsy, Antiepileptic, Anticonvulsant, Tonic-clonic seizures, Clinical field trial

## Abstract

**Background:**

Imepitoin is a novel antiepileptic drug for the treatment of canine idiopathic epilepsy. The present study was conducted to demonstrate superior antiepileptic activity of a high dose of 30 mg/kg BID over a low dose of 1 mg/kg BID of imepitoin during 12 weeks of treatment under double blind conditions in a field population of dogs with previously untreated epilepsy. In a consecutive 12 weeks open label follow up (phase 2), all animals received 30 mg/kg BID, to evaluate the persistence of the antiepileptic activity, and to evaluate the effect of a dose step up to 30 mg/kg in the former low-dose animals.

**Results:**

A treatment with 30 mg/kg BID resulted in a significantly greater reduction in monthly seizure frequency relative to baseline data as compared to the 1 mg/kg dose. Both generalized and partial seizures but not cluster seizures were significantly less frequent in the high dose group. The antiepileptic activity was maintained during study phase 2 in the high dose group. An increase to 30 mg/kg BID in the low- dose animals resulted in a significant reduction in generalized and partial seizures, but not cluster seizures. At the end of study phase 2, 32.1 and 46.8 % of dogs of the former high and former low-dose groups respectively, remained free of generalized tonic-clonic seizures.

Imepitoin was well tolerated. The frequency of dogs with any adverse drug reactions was higher in the 30 mg/kg BID dose (59 % vs. 41 %, *p* = 0.041), and the main target organ was the central nervous system (CNS). The occurrence of CNS related adverse reactions was transient and findings were mostly restricted to the first weeks of treatment. No hepatic enzyme increase and no other organ toxicity were observed.

**Conclusion:**

The administration of imepitoin twice daily at a dose of 30 mg/kg results in significant and persistent antiepileptic effects in patients with newly diagnosed epilepsy and generalized tonic-clonic seizures, as observed over a study period of up to 6 months. Imepitoin was well tolerated. Most CNS related adverse drug reactions were transient. Both the antiepileptic activity and the safety profile make the drug suitable for long-term clinical use.

## Background

Recurrent seizures are a common neurological problem in veterinary medicine. Seizures may occur as the result of an acquired brain disorder (symptomatic epilepsy), or for unknown reasons (cryptogenic epilepsy), or be due to genetic causes, in which case it is referred to as idiopathic epilepsy [1]. The prevalence of canine epilepsy was estimated to be 0.5-5 % of all dogs in the general population or a non-referral based population [2–4], and idiopathic epilepsy has been estimated to represent about 60 -70 % of all cases of epilepsy in dogs [5]. In a recent study, the prevalence was found to be 0.62 % in a large UK population with an increased prevalence in Border terriers and German shepherds and reduced prevalence in West Highland white terriers [6], but idiopathic epilepsy can be found in a wide range of breeds [7, 8].

The current treatment of idiopathic epilepsy is unsatisfactory [9]. If treatment is initiated, only 15 % of cases become seizure free and up to 30 % of canine patients do not experience significant seizure frequency reduction with the most commonly used antiepileptic drugs (AEDs), phenobarbital and potassium bromide [9–11]. Many of these patients are euthanized because of the severity of seizures or because of severe side effects from AEDs [12, 13]. This treatment outcome may be related to the limited availability of treatment options for canine epilepsy [11]. In fact, many AEDs, which have been developed for humans are not suitable for treatment in dogs due to inadequate pharmacokinetics or potential for serious adverse effects, neither of which is acceptable [14–16].

Imepitoin (AWD 131–138 or ELB 138; 1-(4-chlorophenyl)-4-morpholino-imidazolin-2-one) is a new AED approved in the European Union for the treatment of canine idiopathic epilepsy. It was initially developed for the treatment of anxiety and epilepsy in man due to both its pronounced antiseizure activity in a large variety of rodent models of epileptic seizures and its anxiolytic activity in predictive rat models, combined with a high tolerability [17]. Due to inter-individual pharmacokinetic variability in man, which was related to smoking-related induction of metabolic enzymes, the human development was terminated despite excellent tolerability [17, 18]. Based on promising findings with imepitoin in a preclinical seizure model in dogs [19], it was decided to develop this drug for the treatment of canine epilepsy. Initial pilot data had indicated that imepitoin was well tolerated in dogs and that antiepileptic effects were observed both in dogs with newly diagnosed epilepsy as monotherapy and in drug-resistant dogs as add-on treatment to phenobarbital [20]. The aim of this study was to confirm the antiepileptic activity and safety of imepitoin in dogs with idiopathic epilepsy in a well-controlled randomized double blind clinical field study. In contrast to a recently published randomized controlled parallel group study, in which imepitoin was compared with phenobarbital [21], the present study used a low-dose comparator design.

## Methods

### Evaluation of clinical efficacy and safety under field conditions

The study was conducted in 2003 and 2004 as a multicenter, randomized, double blind, controlled parallel group clinical field trial with client-owned animals in compliance with Good Clinical Practice (GCP), aimed at demonstrating superiority of imepitoin 30 mg/kg BID over a low-dose of 1 mg/kg imepitoin administered BID for 12 weeks. The choice of the two doses was based on previous studies in dogs to represent a high but well tolerated dose and the lowest dose with potential antiepileptic activity [17, 19, 20]. This first study phase was followed by 12 weeks open label treatment with 30 mg/kg BID imepitoin (phase 2). Ethical approval was obtained from the ethical board at Hannover Regional Council (Bezirksregierung Hannover), file number 509.6-42502-03A186 for part one and 509.6-42502-03A187 for part two of the study, and from all competent local authorities at each participating study center.

### Study design

About 120 dogs with diagnosis of epilepsy with generalized tonic-clonic seizures were planned to be included at a 1:1 randomization in two treatment arms. The sample size was based on the results of our previous pilot study [19, 20]. A sample size of at least 54 animals per group was calculated to be able to detect a difference of at least one seizure per 28 days with a standard deviation of 1.83 seizures per 28 days, significance level 0.05 and power 80 % (*t*-test). Aiming at including patients with idiopathic epilepsy, the study excluded animals with either reactive seizures or progressive intracranial disease. However, magnetic resonance imaging (MRI) and cerebrospinal fluid (CSF) examination was not made mandatory for the diagnosis, making it possible that animals with other than idiopathic epilepsy were also included. The diagnosis of epilepsy was based on a clinical and neurological examination as described by Vandevelde et al., 2001 [22], excluding patients with clinically manifest or suspected non-idiopathic cause. For inclusion, at least one of the following criteria had to be fulfilled, as determined by retrospective baseline evaluation: two to ten generalized tonic-clonic seizures within three months before randomization, one cluster of tonic-clonic seizures within seven days before randomization, or one convulsive status epilepticus within seven days before randomization.

Dogs were specifically excluded for enrolment if their case history included any of the following: any treatment with antiepileptics including phenobarbital, primidone, or benzodiazepines for longer than four days for treatment of seizures within three months before randomization, intracranial disease as causes of seizures (*e.g.*, neoplastic, degenerative or inflammation-related), or more than ten generalized tonic-clonic seizures within three months before randomization. Pregnant or lactating bitches were not included, as were dogs with a history or clinical symptoms of renal, cardiac, gastro-intestinal or other disorders if the condition in the opinion of the investigator would have exposed the dog to an unacceptable risk or compromised the evaluation of the study results. In addition, the owners had to be willing and capable to comply by written consent with the study procedures. The use of any other AED (*e.g.* phenobarbital, primidone, benzodiazepines and bromides) was prohibited during the study. Any other concomitant treatment or change in concomitant treatment, was to be recorded.

After screening, animals were randomized to one of two treatment arms under double blind conditions. In the high-dose arm, dogs received a dose of 30 mg/kg imepitoin twice daily, while in the low-dose arm the dogs received a dose of 1 mg/kg twice daily. In order to administer the required dose and to ensure blinding, a number of tablets containing imepitoin or placebo and one capsule were to be administered. The high-dose animals received tablets of 100 mg or 400 mg strength, plus one capsule containing granulated tablet material to achieve the exact dose of 30 mg/kg imepitoin. The low-dose animals received a respective number of placebo tablets matching in size and appearance to the 100 and 400 mg imepitoin tablets, plus one capsule containing 1 mg/kg imepitoin as granulated tablet material. Both, tablets (verum or placebo) and capsules were dispensed at a local pharmacy, where capsules were custom filled according to the body weight of the individual dog. To ensure randomization, the pharmacist received a prescription from the local study center with the body weight and the randomization number of the patient. Based on a pre-defined randomization list, he assigned the treatment group, prepared the medication, and dispensed it to the patient owner. On each scheduled visit a new prescription was filled, allowing adjustment of the dose to the actual body weight. This procedure ensured that the respective treatment center and the patient owners remained blinded to the treatment. The treatment duration during phase 1 was scheduled to be 84 days. Visits after inclusion were scheduled after 4, 8, and finally after 12 weeks, +/− 7 days.

Prior to enrolment, the investigators, in collaboration with the owners, documented retrospectively the number and type of seizures, cluster seizures, and convulsive status epilepticus within three months before randomization, to evaluate the seizure frequency related inclusion and exclusion criteria and to document the retrospective baseline. A cluster event was defined as the occurrence of more than four generalized tonic-clonic seizures within 24 h from onset of the first seizure within the cluster. If more than one episode of >4 seizures was observed within one 24 h period, the whole seizure activity within this period was counted as one cluster event. A status epilepticus was defined as a state of continuous seizure activity lasting for 30 min or longer or repeated seizures with failure to return to normal. Partial (focal) seizures were not considered for baseline evaluation.

### Safety evaluation and treatment termination

During the study period, the owners had to keep a patient diary for recording of occurrence and type of seizures, adverse events, dates and times of dosing, and potential changes in concomitant medication. At each visit, a physical and neurological examination was performed, and the patient diary entry was reviewed together with the patient owner. The seizure type and frequency, as well as adverse events, were recorded. At the screening visit and at the final visit, blood and urine samples were taken for determination of safety laboratory values including hepatic and renal parameters, electrolytes, total protein, albumin, and glucose, as well as a complete blood count. Dogs that experienced four generalized tonic-clonic seizures, or any cluster seizure or convulsive status epilepticus within the treatment period, or were given prohibited medication were withdrawn from the study for lack of efficacy. Likewise, dogs experiencing unacceptable tolerability related to the treatment, as judged by the investigator or the patient owner, or dogs suffering from concurrent disease not related to the study treatment and judged to interfere with the study conduct, were terminated. Patients could be withdrawn from the study anytime upon withdrawal of the owners’ consent or obvious non-compliance with the study procedures. Withdrawn animals were included in the final data analysis. Randomized animals withdrawn from the study for any reason other than poor tolerability could be replaced.

### Study phase 2: 12 weeks open label follow up

Dogs that had completed the first phase of the study and dogs that had prematurely terminated the first phase for an exit criterion due to lack of efficacy were eligible to enter in the second phase of the study as open-label follow up. In this phase, all dogs were assigned to receive a treatment of 30 mg/kg imepitoin twice daily, but the owners remained blinded to the treatment during phase 1. The duration of this phase was 84 days, with the possibility of continued treatment on a compassionate basis. At the end of day 84 of the second phase of the study, or earlier if any exit criterion was fulfilled, the investigator was to decide whether treatment could be continued with imepitoin or whether the animal had to leave the study to be treated with standard of care. All study procedures in phase 2 were similar as compared to the first phase of the study, and visits were scheduled after 6 weeks and at the end of the treatment period, i.e. after 12 weeks, +/− 7 days. The combined treatment duration in both successive phases of the study was 24 weeks.

### Statistical evaluation of efficacy and safety

For efficacy evaluation, all treated animals with evaluable retrospective baseline seizure frequency for which at least one measurement of the primary efficacy variable was available were included, representing the intent to treat population (ITT). The change in monthly seizure frequency (MSF) recorded during the treatment period and in the retrospective baseline period was taken as the primary efficacy endpoint. To calculate MSF, the retrospective baseline period (seizure history) and the study period were each divided into six equal intervals (“bins”) of 14 days each. All documented generalized tonic-clonic seizures, clusters or status events were assigned to the corresponding bin. Any cluster or status epilepticus was counted as one seizure event. The seizure frequency (seizures per 14 days) in a bin was defined as the total number of seizures in the bin. Monthly seizure frequencies were then calculated on the basis of one month notionally equaling 28 days. All generalized tonic-clonic seizures and clusters were considered; there were no cases of convulsive status epilepticus during the treatment period. The counting procedure was performed under blinded conditions. The change in monthly seizure frequency was then obtained by subtracting the arithmetic mean of all retrospective baseline bins from the mean of all bins in the study period and was called “monthly seizure frequency - change versus baseline”. Negative values thus represent decreased seizure frequencies, and positive values increased frequencies.

The difference between the change in MSF for the high-dose group and the corresponding change for the low-dose group, as well as the differences in mean MSF prior to and at the end of the treatment period, was analyzed using Student’s two-sample *t*-test with pooled standard deviation, using the two-sided model. To evaluate the efficacy during the open label phase of the study, MSF during the open label study was calculated the same way. The difference between the MSF obtained in phase 2 of the study and in phase 1 was evaluated separately for both treatment groups using the two-sample *t*-test for two repeated measurements. The fraction of animals which were free of generalized tonic-clonic seizures was evaluated as a secondary endpoint separately for both study phases and compared between the two dose groups as well as between phase 1 and 2 using the chi-square test for the equality of two independent proportions following the two-sided model. In addition, the total number of seizures occurring during the two study periods were counted and classified as generalized tonic-clonic seizures, clusters, and partial or complex partial seizures. The frequency of the different seizure types was compared using a Normal-theory test for the equality of two independent rates [23].

The safety of the study treatments was assessed on the basis of adverse events (AEs) reported for all cases enrolled in the study which had been administered at least one dose of study medication following classification according to the VeDDRA list of preferred terms of the system organ classes (European Medicines Agency 2004). The chi-square test for the equality of two proportions was used to compare frequencies of adverse events for individual categories. Laboratory data were evaluated based on standard descriptive statistics and were reviewed for conspicuous individual changes.

## Results

Investigators in eight clinical referral centers specialized in veterinary neurology in Germany and in one center in Switzerland enrolled a total of 127 dogs in the study; 66 and 61 dogs were randomized to the high-dose and the low-dose groups, respectively. All animals met all of the inclusion criteria at screening: 55 and 54 dogs in the high- and low-dose groups, respectively, with two to ten tonic-clonic seizures during the 3-month baseline period; 18 and 11 dogs in the high-dose and low-dose groups, respectively, with one cluster of tonic-clonic seizure within the seven days before randomisation; and three and four dogs in the high-dose and low-dose groups, respectively, with one convulsive status epilepticus within seven days before randomization. Some of these dogs fulfilled more than one of these criteria, and none of the dogs met an exclusion criterion. Two dogs of the high-dose and two dogs of the low-dose group were removed from the ITT population. For one dog assigned to the high-dose group, the owner withdrew consent in the absence of any adverse event after the first dose, and one dog in the high-dose group was not included due to having been erroneously included as a replacement of a dog euthanized after a cluster seizure. One dog from the low-dose group was later diagnosed to suffer from a brain tumor, and another dog from the low-dose group had no available baseline seizure documentation, preventing evaluation of this dog for primary efficacy, although the inclusion criteria were met. However, this dog was included in the evaluation of secondary efficacy parameters.

Dogs from 60 different breeds were included with mixed breed (18) having the greatest frequency, followed by Golden Retriever (8), Labrador Retriever (7), German Shepherd (7), Jack Russell Terrier (6), and various other breeds, mostly with only one representative. While more male than female dogs were included, the treatment groups did not differ in their age and body weight (see Table 1).

**Table 1:** Demography, ITT population of first (blinded) phase of the study

	High dose *N* = 64	Low dose *N* = 60 (59^a^)	Total *N* = 124 (123^a^)
Sex (n) male	41	38	79
female	23	22 (21^a^)	45 (44^a^)
Age [years] Mean ± SD	3.9 ± 2.8	4.4 ± 2.7	4.1 ± 2.8
Range	0 - 13	0 - 13	0 - 13
Weight [Kg] Mean ± SD	26.3 ± 13.0	24.6 ± 13.9	25.4 ± 13.4
Range	3 - 65	2 - 74	2 - 74

^a^For one animal no baseline data were available. It could therefore not be included in the primary efficacy evaluation

In all, 29 dogs (45 %) in the high-dose group and 30 dogs (50 %) in the low-dose group completed the full 12-week study treatment. The other dogs were either withdrawn prematurely or ceased treatment early because an exit criterion was fulfilled. In the high-dose group, the occurrence of the 4th generalized tonic-clonic seizure or a cluster seizure (10 and 17 cases, respectively) was the most frequent exit criterion, while suspected adverse reactions (4), consent withdrawal (3) and non-compliance (1 case) were rare reasons. In the low-dose group, a similar distribution was seen, with 4th generalized tonic-clonic seizure seen in 13 cases and cluster seizure in 11 cases being most frequent. Suspected adverse reactions (1), consent withdrawal (2) and non-compliance (3) were rare reasons for premature discontinuation. All animals that completed the treatment period or were withdrawn prematurely for lack of efficacy were allowed to continue in the open label phase of the trial. This procedure was selected to enable the transition of the low-dose animals to high-dose treatment without having to break the blind. Consequently, 53 out of 56 eligible dogs of the former high-dose group continued with the open label treatment, while 47 of 53 eligible dogs of the low-dose group transitioned to the open label phase and were started on 30 mg/kg BID. In the remaining dogs, the owners withdrew consent for the 2^nd^ phase of the study.

### Antiepileptic activity

**Fig. 1:**
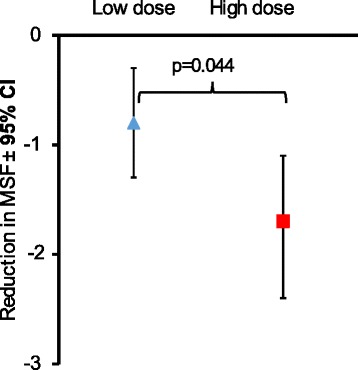
Reduction in monthly seizure frequency (MSF) during study phase 1. Displayed are mean ± SD of the difference in MSF prior to start of treatment (retrospective baseline) and during treatment with 1 mg/kg BID (Low dose) or 30 mg/kg BID (high dose). The MSF was significantly more reduced in the high dose group (two-sided two-sample *t*-test)

The evaluation of the baseline seizure frequency of generalized tonic-clonic seizures, cluster seizures or status epilepticus events revealed that, in the high-dose group, despite randomized inclusion, the mean seizure frequency was found to be higher than in the low- dose group. This difference was statistically significant (*p* = 0.030). In both groups, the mean seizure frequency dropped during the study period, but the drop was more pronounced in the high-dose group, reaching a reduction of 1.7 ± 2.8 seizures per month, while the drop in seizure frequency was only 0.8 ± 2.0 seizures per month in the low-dose group. This mean difference was statistically significant (*p* = 0.044), indicating that the high dose was superior to the low dose in reducing seizure frequency (Table 2 and Fig. 1). In the high-dose group, 37.5 % of animals (24 of 64) became seizure free, but also in the low-dose group 31.7 % (19 of 60) became seizure free, the difference was not significant. In addition, the total number of different seizure types which occurred during the first study phase was counted and the rate of seizures per dog was calculated. This included also counting of partial and complex partial seizures, in addition to generalized tonic-clonic and cluster seizures (Table 3). Both groups differed significantly (*p* = 0.035) in the rate of generalized tonic-clonic seizures, but not in cluster seizures. The frequency of partial and complex partial seizures was also significantly lower in the high-dose group (*p* < 0.001). When all seizures were summed, the total number of seizures was also significantly lower in the high-dose group (*p* < 0.001).

**Table 2:** Monthly seizure frequency (MSF) and proportion of seizure free animals during phase 1 and 2

	High-dose *N* = 64	Low-Dose *N* = 60 (59^a^)	Significance^b^, high-dose vs. low-dose group
ᅟMSF Baseline Mean ± SD	2.9 ± 2.7	2.0 ± 1.7	*p* = 0.030
ᅟ95 CI of mean	2.2 - 3.6	1.6 – 2.5	
ᅟMSF During phase 1 Mean ± SD	1.2 ± 1.7	1.3 ± 1.8	*p* = 0.751
ᅟ95 CI of mean	0.7 - 1.6	0.8 - 1.7	
ᅟMSF Change to baseline Mean ± SD	−1.7 ± 2.8	−0.8 ± 2.0	*p* = 0.044
ᅟ95 CI of mean	−2.4 - -1.1	−1.3 - -0.3	
ᅟSeizure free animals during phase 1 [n]	24 of 64	19 of 60	*p =* 0.495
Transition to phase 2	*N* = 53 of 64	*N* = 47 of 60	
ᅟMSF During phase 2 Mean ± SD	1.2 ± 1.3	0.9 ± 1.4	*p =* 0.269
ᅟ95 CI of mean	0.8 - 1.5 *p =* 0.688^c^	0.5 - 1.4 *p* =0.369^c^	
ᅟSeizure free animals at start of phase 2 [n]	16 of 53	14 of 47	*p* = 0.965
ᅟSeizure free animals during phase 2 [n]	17 of 53 *p* = 0.540^c^	22 of 47 *p* = 0.110^c^	*p* = 0.132

^a^For one animal no baseline data were available. It could therefore not be included in the primary efficacy evaluation

^b^The *p*-value for the difference in mean MSF and change in MSF was based on the two-sided two-sample *t*-test; the *p*-value difference in the proportion of seizure free animals was based on the two-sided chi-square test for two samples

^c^Comparison of results from phase 1 and phase 2 of the study

**Table 3:** Number of different seizure types and average seizure rate per animal

		High dose (30 mg/kg)	Low dose (1 mg/kg) High dose in Phase 2	Significance^a^, high dose vs. low dose group
	Seizure type	Total number (rate)	Total number (rate)	
Phase 1: Primary Efficacy Evaluation	Number of animals in phase 1	*N* = 64	*N* = 60	
	Generalized tonic-clonic seizures	73 (1.14)	95 (1.58)	*p* = 0.035
	Cluster seizures	17 (0.27)	9 (0.15)	*p* = 0.156
	Partial or complex partial seizures	30 (0.47)	75 (1.25)	*p* < 0.001
	Total during part 1	120 (1.88)	179 (2.98)	*p* < 0.001
Phase 2: Extended use at high dose	Number of animals in phase 2	*N* = 53	*N* = 47	
	Generalized tonic-clonic	60 (1.13) *p* = 0.966^b^	45 (0.96) *p *= 0.004^b^	*p* = 0.393
	Cluster seizures, convulsive status	21 (0.40) *p* = 0.226^b^	9 (0.19) *p* = 0.609^b^	*p* = 0.057
	Partial or complex partial seizures	5 (0.09) *p* < 0.001^b^	19 (0.40) *p* < 0.001^b^	*p* = 0.002
	Total during phase 2	86 (1.62) *p* = 0.303^b^ vs. phase 1	73 (1.55) *p* = 0.001^b^	*p* = 0.783

^a^Based on the Normal-theory test for the difference in rates between two independent samples [23]

^b^Comparison of results from phase 1 and phase 2 of the study

In total 100 animals, 53 of the high-dose group and 47 of the low-dose group continued open label treatment in phase 2 with BID 30 mg/kg imepitoin. There was no difference in MSF in the high-dose animals comparing both study phases, indicating continued antiepileptic activity. The reduction obtained during study initiation was maintained with a mean MSF of 1.2 ± 1.3 in phase 2. In the animals of the former low-dose arm, the mean MSF in phase 1 of the study was already low with 1.3 ± 1.8 seizures per month. The increase in dose to 30 mg/kg BID resulted in a slight, but not significant, reduction in mean MSF to 0.9 ± 1.4 seizures per month. However, the dose increase resulted in an increased number of seizure free dogs. Considering only the dogs which migrated to the 2^nd^ study phase, 30.2 % (16 of 53 dogs) of the former high-dose group and 29.8 % (14 of 47 dogs) of the former low-dose group were seizure free at the start of phase 2. During the 12 weeks open label treatment, 32.1 % (17 of 53) of the former high-dose animals were seizure free, while in the former low- dose group the increase in dose resulted in a rise in seizure free animals to 46.8 % (22 of 47 dogs). This increase in the number of seizure free animals (8) during phase 2 for the former low-dose animals, although just not statistically significant (*p* = 0.055, one sided, or *p* =0.110, two sided Chi-square test), is a noteworthy finding (Table 2).

The total number of different seizure types which occurred during the second study phase was counted, and this included again also partial and complex partial seizures (Table 3). The two groups did not differ significantly in the number of generalized tonic-clonic seizures, cluster seizures, and all seizures, as may be expected in that both groups were dosed with 30 mg/kg BID in this study phase. However, if the study phases 1 and 2 were compared for the former low-dose group, it became evident that the rates of generalized tonic-clonic seizures, partial or complex partial seizures, and of all seizures were significantly reduced, while the rate of cluster seizures was not different (Table 3). In contrast, in the former high-dose group, which was continued on 30 mg/kg BID, there was no significant change in seizure rate with the exception of partial seizures, which were further reduced (p < 0.001) (Table 3). These data indicate, that the antiepileptic activity reached in phase 1 of the study was maintained in phase 2.

### Safety

The safety evaluation included all 127 cases that had obtained at least one dose of active drug. At least one adverse event (AE) was observed for 86 % of the high-dose animals, and for 82 % of the low-dose group animals during study phase 1. The total number of reports was 213 in the high-dose group and 168 in the low-dose group. The number of reports of AEs in the high-dose group was greater than in the low-dose group, but the numbers of dogs affected were not substantially different between the two groups (*p* = 0.497 for any event). If only the dogs with AEs which were judged to be likely treatment related, *i.e.* which were classified as adverse reactions (ARs) were concerned, the high-dose animals were significantly more frequently affected (*p* = 0.041). The incidence of dogs affected by AEs and ARs in each body system was generally about the same in the two groups, with the following three exceptions. There were significantly more dogs with CNS-related AEs in the high-dose group (*p* = 0.018), and more of these were rated as ARs (*p* = 0.031). These ARs were predominantly ataxia, disorientation (and disturbance of equilibrium or co-ordination), hyperactivity, and restlessness. There was a trend for a higher rate of ARs for the musculoskeletal system in the high-dose compared to the low-dose group, but the difference did not reach level of significance (*p* = 0.057, Table 4). These were largely related to motor activity, and thus overlap with hyperactivity and restlessness. Most AEs were mild or moderate. Gastrointestinal events were relatively frequent in both groups, but no relationship to the dose was seen, so these may be assumed not to have been caused by the study treatment. In most cases, the AEs were transient and observed primarily during only the first weeks of treatment; this applied especially to the AEs observed most often in the high-dose group, *e.g.*, ataxia and disorientation, as well as hyperactivity (details not shown).

**Table 4:** Summary of most frequent adverse events by organ system, number of dogs affected

Category of adverse events	High dose, *N* = 66 Number of dogs (dogs with likely treatment relation)	Low dose, *N* = 61 Number of dogs (dogs with likely treatment relation)	Significance^a^, high vs. low-dose group (dogs with likely treatment relation)
Any	57 (39)	50 (25)	*p* = 0.497 (*p* = 0.041)
Central nervous system^b^	48 (33^a^)	32 (19^a^)	*p* = 0.018 (*p* = 0.031)
Gastro-intestinal system	32 (8)	25 (2)	*p* = 0.396 (*p* = 0.065)
Musculoskeletal system	12 (10)	8 (3)	*p* = 0.443 (*p* = 0.057)
Respiratory system	7 (4)	8 (4)	*p* = 0.662 (*p* = 0.908)
General	5 (2)	5 (2)	*p* = 0.897 (*p* = 0.936)
Urogenital tract	4 (1)	5 (1)	*p* = 0.639 (*p* = 0.956)
Other systems	14 (10)	15 (3)	*p* = 0.650 (*p* = 0.057)

^a^Based on the two-sided chi-square test for the equality of proportions from two independent samples

^b^Mainly seen during first weeks of treatment

In phase 2 of the study, the total number of reports of adverse events was 208 (95 in dogs from the former high-dose group and 113 in dogs from the former low-dose group. The frequency of reports and a likely relationship to the study treatment as rated by the investigator were somewhat higher in dogs from the former low-dose group, but no significant difference could be seen between both groups. The distribution of AEs by organ systems was similar to the findings in phase 1, including a relatively high frequency of CNS-related events and the transient nature of the CNS-related events (data not shown).

At the time of enrolment of the dogs in the study, there were no significant differences between the treatment groups with respect to the blood chemistry indicators including markers of hepatic and renal function, and in both treatment groups, similar frequencies of animals with individual values outside the reference values of the analytical laboratory were found during visit one. At the end of study phase 1, blood chemistry indicators were in general unchanged for both groups; some values (hepatic values: alanine aminotransferase, alkaline phosphatase, glutamate dehydrogenase) even improved during the study in both groups (data not shown). There was a slight tendency that creatinine values increased in the high-dose group from 84.9 ± 21.5 μmol/l to 99.5 ± 26.4 μmol/l, while the change was minimal in the low-dose group from 82.2 ± 21.0 to 84.0 ± 19.9 μmol/l at the end of study phase 1. During study phase 2, the creatinine value did not change further in the high-dose group (96.4 ± 31.0 μmol/l), while in the former low-dose group the creatinine levels slightly increased to 91.9 ± 25.8 μmol/l. However, all measurements remained within the normal range. Hematological values were stable in both groups during both study periods (data not shown).

## Discussion

The purpose of the current study was to demonstrate antiepileptic activity and safety of imepitoin in dogs with generalized tonic-clonic seizures suffering from idiopathic epilepsy. The diagnosis was primarily based on seizure history in combination with a normal clinical and neurological examination as well as normal laboratory data to exclude extracerebral causes of seizures or progressive intracranial disease such as neoplasia or inflammation. This procedure does not preclude the inclusion of patients with other than idiopathic epilepsy, such as patients with cryptogenic or symptomatic epilepsy. The prevalence of clinically significant MRI abnormalities in dogs below the age of 6 years with seizures without interictal neurological deficits was previously found to be one out of 46 dogs [24], indicating that the vast majority of the dogs included in this study, which were aged 3.9 ± 2.8 and 4.4 ± 2.7 years, had indeed idiopathic epilepsy. Only 26 of 127 dogs were aged above 6 years, with 10 each being 7 and 8 years old at inclusion. However, it cannot be excluded that individual dogs with undetermined causes of epilepsy had been also included. Indeed, one dog was excluded from efficacy evaluation prior to unblinding due to other causes of seizures determined by MR imaging.

The optimal study design to demonstrate a clinical effect in a controlled field study is to conduct a randomized double blind placebo controlled study with a sufficiently long prospective baseline to establish an exact measure of baseline seizure activity. However, a seizure disease presents with massive behavioral manifestations, and if patient owners seek help for treatment of this disease, a placebo controlled study and a prospective baseline was at the time of the study conduct ethically questionable. Add-on trials to established antiepileptic drugs in patients with drug resistant epilepsy has been the primary approach for many years in human medicine. Because co-administered drugs are subject to drug interactions, add-on trials of antiepileptic drugs do not necessarily address the utility of a new drug as monotherapy or its use in patients with newly diagnosed epilepsy [25]. A low-dose active comparator design was therefore developed to enable demonstration of clinical efficacy of a new drug using a superiority design [25]. We have selected and adapted this design principle and have compared a low dose of 1 mg/kg BID of imepitoin with the high dose of 30 mg/kg BID, involving a double-blind placebo-controlled parallel group study design with retrospective baseline. While the retrospective baseline did introduce possibility for inaccurate recording of seizure activity, this was minimized by focusing on generalized tonic-clonic seizures, clusters, and status epilepticus events, which, due to their severity, can be well memorized and counted. The selection of the low dose was based on a previous pilot study and on preclinical data in seizure models. In an open label pilot study, first antiepileptic effects were seen at a starting dose of 5 mg/kg BID [19, 20]. Experimental data indicated that doses as low as 1 mg/kg of imepitoin were capable of elevating the seizure threshold in the amygdala kindling model of epilepsy in rats [17], and with a dose of 5 mg/kg administered once daily for up to 40 days, the threshold for induction of seizures was significantly elevated in dogs [19]. Based on these data, the dose of 1 mg/kg, administered BID, was selected as the potential lowest dose with antiepileptic activity. The high dose was selected from the highest dose tested in the pilot study, which was also found to be safe [19, 20]. The study design developed consisting of the described two treatment phases enabled the evaluation of antiepileptic activity over a total treatment period of 6 months in dogs, which were continued at the high dose, and in fact, the antiepileptic activity obtained during the first 12 weeks was maintained during the 2^nd^ study phase. Moreover, 17 dogs continued treatment under compassionate use and were followed up for up to 2 years, maintaining antiepileptic activity (data not shown).

In this study, we focused on previously untreated dogs with epilepsy. The aim was to select a homogenous group of newly diagnosed patients, but the inclusion criteria allowed for both patients with a history of untreated generalized tonic-clonic seizures and also patients with a recent history of a single cluster seizure event or a recent status event. While most patients had a history of generalized tonic-clonic seizures, 29 dogs had one or more clusters of tonic-clonic seizures, and nine dogs had one convulsive status epilepticus within the seven days before randomization. Those seizure events were either the sole event or probably the reason why the owner sought medical treatment. Patients which had experienced cluster seizures are known to be significantly less likely to achieve remission upon any antiepileptic treatment [26]. Our inclusion criteria allowed therefore the inclusion of potentially drug resistant dogs. By chance, 18 animals with clusters were included in the high-dose group, while only nine animals with clusters were included in the low-dose group during randomization. In addition, MSF at baseline was found to be significantly higher in the high-dose group, as compared to the low-dose group (Table 2). However, since the individual difference in MSF during baseline and during study phase 1 was used to evaluate clinical efficacy, this baseline difference had little influence on the primary efficacy outcome. MSF in animals treated with the high dose was significantly more reduced than in the low-dose group, indicating that a high dose of imepitoin is superior to a low dose in reducing the monthly frequency of generalized tonic-clonic seizures.

The MSF was also reduced in the low-dose arm, and even 19 of 60 dogs evaluated were free of generalized seizures during study phase 1. While one may conclude that the dose of 1 mg/kg given BID may be indeed effective in at least a subset of dogs, other factors need to be considered. The seizure frequency in epileptic diseases fluctuates over time, with times of higher frequency, and times with fewer seizures, as has been shown in childhood epilepsy [27]. If owners of previously untreated patients are seeking treatment, one may reasonably assume that this decision was guided by a recent experience of seizure events, potentially marking a period of increased seizure activity. As soon as treatment, including placebo treatment, is initiated, the period of increased activity may be followed spontaneously by a period with reduced seizure activity, without causal relation to the treatment [27]. This phenomenon is called regression to the mean [28, 29]. The Hawthorne-effect, i.e. the change in behavior due to the fact that a patient (or the patient owner) is participating in a clinical study, and the placebo effect, which is induced by the fact that a patient (or in case of pets the pet owner) may expect a pharmacological effect from a new treatment, may also contribute to the reduction in seizure frequency in the low-dose or placebo group [30]. Burneo et al. [29] conducted a meta-analysis of placebo controlled trials in epilepsy in man. They found, that due to all three factors, 9.3-16.6 % of patients in the placebo arm had a >50 % reduction in seizure frequency. This effect represented 20-50 % of the activity observed for the active agents. It is therefore likely that at least a proportion of the reduction in MSF may not be linked to pharmacological activity of the 1 mg/kg BID dose. In a seizure threshold model in dogs, significant effects were seen for an oral dose of 5 mg/kg administered once daily if the seizure event was induced at the time of peak plasma level, indicating that low doses can have an antiepileptic effect [19]. While lower doses than 5 mg/kg have not been tested in this model, the small threshold effect observed at 5 mg/kg makes it unlikely that a dose of 1 mg/kg can have sufficient antiepileptic activity to solely explain the MSF reduction in the low-dose group. A meta-analysis of three placebo-controlled clinical trials in epileptic dogs could verify a placebo response in dogs with epilepsy [31], and a clear placebo response was also seen in a study testing levetiracetam using a cross over design [32].

The percentage of patients which were free of generalized tonic-clonic seizures was evaluated as a secondary endpoint. In the high-dose group, 37.5 % and in the low-dose group 31.7 % of all animals were seizure free during the first study phase. The lack of a significant difference between the two treatment groups is likely due to the difference in baseline seizure activity, which was higher in the high-dose group. In the animals from the low-dose group which were treated with the high dose in the 2^nd^ phase of the study, the number of animals which were free of generalized tonic-clonic seizures reached 46.8 % (22 of 47 dogs), up from 29.8 % at start of phase 2, indicating increased activity of the high dose over the low dose, while in the high-dose animals which were maintained on high dose the fraction of seizure free animals was 32.1 %, indicating stable antiepileptic activity. In a recent review of 344 dogs undergoing antiepileptic treatment of any kind in a referral clinic, only 14 % became seizure free [26], indicating that imepitoin is very efficacious in responsive patients. While the number of seizure-free dogs increased in the former low-dose group during study phase 2, MSF was only slightly further lowered in this group (Table 2). This may be related to the fact that about one third of animals has been reported to remain treatment resistant with any treatment [11], and thus this fraction of animals did not improve with imepitoin.

Generalized tonic-clonic seizures (as well as cluster seizures or status epilepticus events) are conditions which are disruptive for the patient owner as well as for the patient. We focused on these seizure types to define the efficacy of imepitoin. These seizures could be also safely classified for the retrospective seizure history [33]. During the treatment periods, the owners were required to keep a daily diary and were educated to identify and record also partial and complex partial seizure activity. The treatment with imepitoin at 30 mg/kg BID resulted in a significant reduction in all seizure types except for cluster seizures compared to the low-dose treatment in phase 1, and upon continuation in phase 2, all seizure types except for cluster seizures were significantly reduced when the dose was increased from 1 mg to 30 mg/kg BID in phase 2. Partial and complex partial seizures were most strongly reduced in the high-dose group as compared to the low-dose group during study phase 1, and when the low-dose animals were dosed with the high dose in the 2^nd^ study phase, the number of partial seizures was also significantly reduced, indicating that imepitoin may have a potent effect against these seizure types (Table 3). Imepitoin is a low affinity partial agonist at the benzodiazepine receptor [17]. Benzodiazepines are long known to be active against diverse seizure types including partial seizures, complex partial seizures, absence seizures, and myoclonic seizures, in addition to generalized tonic- clonic seizures [34, 35]. The interesting activity of imepitoin against partial and complex partial seizures may therefore be related to its mode of action.

Antiepileptic treatment requires chronic medication. While benzodiazepines are generally known to be potent antiepileptics, their clinical use in man is limited by the development of tolerance to the antiepileptic activity [34]. In various experimental models imepitoin has not been shown to be susceptible to tolerance development [17, 19]. We now demonstrate that, in a clinical field setting, no tolerance developed to the antiepileptic activity of imepitoin during continuous treatment for up to 6 months under field conditions. Imepitoin may therefore be used for long-term treatment without risk of losing antiepileptic activity. The suitability for long-term use is also supported by the safety profile of imepitoin as found in this study. The most frequent organ systems with an observed adverse event were the CNS, gastrointestinal system, and musculoskeletal system; however, only few findings in the gastrointestinal system were judged to be treatment related by the investigator. The CNS and musculoskeletal system related ARs reported in this study, *i.e.* ataxia, disorientation, hyperactivity, and restlessness, are similar to the ARs reported in a previously reported clinical study [21]. It is however interesting to note, that these findings were reported by the investigators to be transient. The possible treatment relation of these CNS related findings is supported by the fact, that in the 2^nd^ phase of the study, the frequency of these findings increased in the dogs which had been previously dosed with the low dose. Again, the investigators reported that these findings were predominantly in the first weeks of treatment with the high dose. The transient nature of the CNS related ARs has been also reported in the pilot study of imepitoin [19, 20]. The safety profile of imepitoin differs markedly from phenobarbital, which has been found to induce significantly more CNS-related symptoms including somnolence/sedation and neurological disorders, but also significantly more other ARs, including increased appetite, polydipsia, polyuria, renal/urinary disorders and diarrhea [21]. Only hyperactivity had been found to be significantly more frequent for the imepitoin group [21]. Our study results now indicate that these findings are transient for imepitoin.

Both, clinical biochemistry and hematology investigations did not reveal any clinically relevant ARs. Liver enzymes were not increased and, in phase 1, were even reduced -- again in contrast to the findings observed for phenobarbital [21]. A marginal increase in mean creatinine was observed in our study, and the treatment relation could be confirmed by the fact that the same increase was seen in study phase 2 in dogs of the former low-dose group. In dogs which were maintained on the high dose, no further increase in creatinine was seen. No other changes in kidney related safety parameters were seen. Because only a marked damage of functioning nephrons can result in increased creatinine levels in serum [36], and because such damage was not seen even following dosing of 150 mg/kg imepitoin for 6 months [21], the increased serum creatinine level may be a result of increased muscular turnover and not an indicator of kidney dysfunction. An alternative explanation to the slight but discrete increase in creatinine may be related to the analytical method used. Indeed, the standard method is highly susceptible to interference by many factors, including drugs and metabolites [37].

## Conclusion

Based on the results of this clinical randomized controlled field study the administration of imepitoin twice daily at a dose of 30 mg/kg results in significant and persistent antiepileptic effects in patients with newly diagnosed epilepsy suffering from generalized tonic-clonic seizures, as observed over a study period of up to 6 months. The safety profile of imepitoin was good, and mostly CNS related ARs were transient and predominantly observed in the first weeks of treatment. Both, the antiepileptic activity and the safety profile lacking hepatotoxicity or other organ toxicity make the drug suitable for long-term clinical use at the recommended therapeutic dose.

## Abbreviations

AE: Adverse event (all)
AED: Antiepileptic drug
AR: Adverse reaction (with possible or likely treatment relation)
BID: Twice daily
CNS: Central nervous system
GCP: Good clinical practice
ITT: Intent to treat population
MSF: Monthly seizure frequency
